# Evaluation of Green Tea Leaves as an In Situ Capping Material for the Remediation of Lindane-Contaminated Sediments

**DOI:** 10.1021/acsomega.5c01779

**Published:** 2025-07-29

**Authors:** Chi-Wei Wang, Chenju Liang

**Affiliations:** † Department of Environmental Engineering, 34916 National Chung Hsing University, 145 Xingda Rd., South Dist., Taichung 402202, Taiwan; ‡ Department of Environmental Engineering, Da-Yeh University, 168 University Rd., Dacun, Changhua 515006, Taiwan

## Abstract

Organochlorine pesticides (OCPs), such as lindane, persist as toxic pollutants in river sediments, necessitating operative and effective remediation. An alkaline green tea (GT)/Fe^2+^ system shows promise for chemical reductive degradation of lindane through sustained polyphenol release. This study developed a novel in situ capping material (ISCM) composed of tea leaves, bentonite, sodium alginate, and pyrite. The GT ISCM exhibits strong water absorption, swelling, extended polyphenol release, and a stable reducing environment. Using the Taguchi method (L9 orthogonal array), the ISCM formulation was systematically optimized, and Analysis of Variance identified the optimal composition as 10 g bentonite, 0.5 g tea leaves, 0.25 g pyrite, and 0.1 g sodium alginate. Utilizing tea polyphenols as reducing agents, it immobilized lindane and promoted its degradation while preventing the formation of 1,3,4,5,6-pentachlorocyclohexene and trichlorobenzene, byproducts produced during alkaline hydrolysis. Under simulated field conditions, the ISCM significantly reduced lindane release from contaminated sediments into the aqueous phase, demonstrating high removal efficiencies. These findings underscore the potential of GT ISCM as a sustainable strategy for stabilizing and degrading lindane in contaminated sediments, providing a green alternative for OCP remediation.

## Graphical abstract

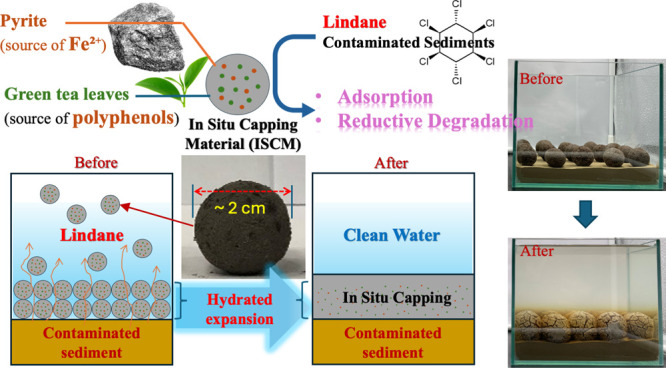

## 1 Introduction

Organochlorine pesticides (OCPs), among the first synthetic insecticides for agriculture, were widely adopted from the 1940s due to their stability, persistence, and effectiveness.^1^ In Taiwan, OCPs such as lindane (γ-hexachlorocyclohexane) were heavily used in the 1950s.^2^ Moreover, Doong et al.^2^ found total hexachlorocyclohexane concentrations in Taiwanese river sediments ranging from 0.57 to 14.1 ng g^–1^. The World Health Organization reports that lindane remains detectable in surface waters at 0.01–0.1 mg L^–1^.^3^ As lindane degrades in a natural environment, it undergoes dehydrochlorination to form 1,3,4,5,6-pentachlorocyclohexene (PCCHe), which further breaks down into toxic trichlorobenzene (TCB) isomers.^4–6^ TCBs are known to harm the liver, kidneys, and adrenal glands.^7^ Consequently, the development of effective remediation strategies for OCP-contaminated sediments remains a critical environmental priority.

In ecological systems, sediments play crucial roles, including facilitating self-purification processes and providing habitats for diverse organisms. However, sediments contaminated by POPs pose substantial ecological and environmental risks, as these pollutants can migrate through the food chain, adversely affecting plants, animals, and human health. Sediment remediation presents unique challenges due to its submerged location in rivers and lakes. Key obstacles include ensuring the effective delivery and prolonged stability of remediation reagents in expansive water bodies, while also counteracting the destabilizing effects of river currents on reactive capping materials.^8^ Sediment remediation strategies currently include dredging, confined disposal facilities, monitored natural recovery, confined aquatic disposal, and in situ capping (ISC).^9^ Among these, ISC has gained prominence in recent years due to its effectiveness and relatively minimal environmental disturbance.

The principle of ISC involves covering contaminated sediments with clean or reactive materials to reduce the bioavailability of pollutants and slow their migration and dispersion. This technique may utilize geotextile liners and other permeable or impermeable materials, and often incorporates amendments such as organic carbon within composite layers to further reduce pollutant mobility. For instance, Murphy et al.^10^ demonstrated the use of activated carbon (AC) and zeolites, known for their adsorption properties, as capping materials to enhance the stabilization of sediments contaminated with 2,4,5-polychlorinated biphenyls. Additionally, various high-surface-area adsorbents, such as clays, fly ash, and others or those combined with carbon-based materials like black carbon, carbon nanotubes, or biochar, may also be considered as potential capping materials.^11^ The combination of adsorption and chemical degradation has proven to be an effective remediation strategy, particularly when employing reductive processes involving iron sulfide^11^ or ferrous ions.^12^ Similarly, Zimmerman et al.^13^ investigated the effects of covering polychlorinated biphenyl (PCB)-contaminated sediments with layers of gravel and AC, finding that AC retained more than 89% of PCB within the contaminated sediments. Zang et al.^14^ combined calcium peroxide with zerovalent iron to enhance in situ sediment remediation, enabling simultaneous denitrification and phosphorus stabilization. This approach is an example of active capping, which broadly refers to sediment caps that interact with contaminants through processes such as adsorption, sequestration, or degradation. In their study, degradation is the primary mechanism involved. Their results showed that the system removed approximately 31–44% of organic matter from the sediments.^15,16^ However, because sediments lie submerged at the bottom of rivers and lakes beneath large volumes of water, effectively remediating them without disturbing the sediment layer remains a significant challenge. Thus, the development of effective reactive materials and optimized implementation techniques for active capping holds substantial potential for addressing sediment contamination.

Natural antioxidant polyphenols, such as flavonoids, encompass over 4000 distinct compounds and are widely found in plants, particularly in green leaves and fruit skins.^17^ Tea leaves are abundant in flavonoids, particularly catechins such as epicatechin (EC), epigallocatechin (EGC), epicatechin gallate (ECG), and epigallocatechin gallate (EGCG), which are among the most notable.^18^ Polyphenols possess strong reducing abilities because of the phenolic hydroxyl groups in their structure, with the strength of this ability dependent on the number and arrangement of hydroxyl groups (−OH). As antioxidants, polyphenols must meet two key conditions: first, at concentrations lower than that of the substrate to be oxidized, they must prevent auto-oxidation or free radical-mediated oxidation; second, the intermediate radicals formed after scavenging free radicals must stabilize through further oxidation via intramolecular hydrogen bonding, during which electrons are released.^19,20^ Depending on their structure and redox properties, polyphenols can act as reducing agents, hydrogen donors, free radical scavengers, and metal ion chelators.^17^ In studies by Liang’s research group,^18,21^ it was reported that under alkaline conditions (pH > 10), green tea extract (GTE) combined with Fe^2+^ effectively degraded lindane in aqueous solution. Because most polyphenols have dissociation constant (p*K*_a_) above 7,^22^ they readily deprotonate under alkaline conditions, releasing hydrogen ions and donating electrons.^23–25^ Moreover, the hydroxyl groups in the catechins within GTE played a crucial role in reducing Fe^3+^, thus preventing Fe^2+^ from oxidizing in an alkaline environment and maintaining an electron-rich reducing environment. When green tea leaves were directly added to the reaction system, significant lindane adsorption and subsequent degradation were observed. These findings indicated that this green tea leaves/iron system has significant potential as a multifunctional agent for environmental remediation. Under alkaline conditions, neither polyphenols nor ferrous ions alone significantly reduce lindane. Polyphenols alone mainly trigger alkaline hydrolysis, producing more toxic trichlorobenzene isomers.^18^ However, when combined, tea-derived polyphenols and ferrous ions form a complex that enhances electron transfer and supports Fe^2+^/Fe^3+^ redox cycling. This synergy promotes reductive dechlorination, making the system more effective than either component alone. Building on this mechanism, the present study integrated the polyphenol-Fe^2+^ system into an in situ capping approach to achieve both pollutant containment and chemical transformation in contaminated sediments.

To extend the conventional ISC technique beyond mere sediment stabilization to also actively degrade pollutants, this study developed an in situ capping material (ISCM) enriched with polyphenolic substances from green tea leaves for remediating lindane-contaminated sediments. Harnessing the reductive properties of tea leaves, the composite ISCM was prepared using fresh green tea leaves, ferrous ion bearing pyrite, bentonite, and sodium alginate as encapsulating agents,^26,27^ allowing the ISCM to swell upon application and form a reactive capping layer over the sediment surface. This capping layer can serve as a physical barrier, stabilize sediments, and reductively degrade lindane. The objectives of this study were: (1) to design ISCM formulations using the Taguchi method and conduct characterization analyses; (2) to assess the potential of the ISCM for degrading lindane in an aqueous system; and (3) to evaluate its potential application in remediating contaminated sediments.

## 2 Materials and Methods

### 2.1 Chemicals

Chemicals used were purchased from the following sources: Ferrous sulfate (FeSO_4_·7H_2_O, ≥99%) was purchased from Union Chemical Works Ltd. Lindane (C_6_H_6_Cl_6_, 99.9%) was purchased from Fluka. Acetone (CH_3_COCH_3_, ≥99.5%) was purchased from J.T. Baker. *n*-Hexane (C_6_H_14_, 95%) was purchased from Tedia. Natural mineral pyrite (FeS_2_, 97%, 0.21–0.60 mm) was purchased from a local mining company (Ruhnyu, Inc., Tainan, Taiwan). Folin & Ciocalteu’s phenol reagent, alginate acid sodium, and bentonite were purchased from Sigma-Aldrich. Commercial green tea leaf was purchased from Taiwan Ten Ren Tea Co., Ltd. The water used was purified by a reverse osmosis (RO) purification system (Sky Water XL-300A).

### 2.2 Experimental Procedures

#### 2.2.1 ISCM Preparation

Bentonite was selected as the primary material due to its proven application in commercially available sediment capping material AquaBlok,^28^ which is known for its high water absorption capacity, enhancing the physical properties of the ISCM. Sodium alginate was included to prevent cracking during the drying process^29,30^ and to improve the stability of the ISCM when deployed in water. Green tea leaves and pyrite were incorporated to provide Fe^2+^ for the polyphenol/Fe^2+^ reaction system. Unlike previous studies that directly added ferrous sulfate,^21^ this study utilized pyrite as the Fe^2+^ source to prevent the rapid release of iron ions into water. Such rapid release could interfere with the water absorption and swelling of bentonite, potentially causing disintegration.^31^ Additionally, the weight of pyrite facilitated the rapid settling of the ISCM onto the sediment surface.

The ISCM used in this study was composed of bentonite, sodium alginate, green tea leaves, and pyrite. The preparation process involved thoroughly mixing appropriate amounts of bentonite, green tea leaves, pyrite powder, and a sodium alginate solution. The mixture was molded into spherical shapes and allowed to dry at air-conditioned temperatures (24–26 °C) for 1 day, resulting in solid spheres and hardened. The ISCM was shaped into spheres to ensure uniformity and mechanical stability during laboratory testing. This configuration enables consistent handling, clear visual observation, and precise placement. In contrast, powdered forms are prone to dispersion and uneven settling under hydrodynamic conditions. Therefore, the spherical design was chosen to enhance experimental reliability and better reflect practical application scenarios. The Taguchi method was employed to design an orthogonal array for testing different combinations of the four components (bentonite, sodium alginate, green tea leaves, and pyrite) at three dosage levels. The Taguchi method is a statistical approach that optimizes experimental conditions and improves quality performance with a reduced number of experiments. In this study, a Taguchi L9 (3^4^) orthogonal array was used to evaluate the effects of the four factors. The experimental design, detailing the four factors and three levels for each material, is presented in Table 1.

**Table 1:** L9 (3^4^) Orthogonal Array Experimental Design

		materials			
levels		bentonite (g)	tea leaves (g)	pyrite (g)	sodium alginate (g)
1		5	0.125	0.25	0.025
2		7.5	0.25	0.5	0.05
3		10	0.5	1.0	0.1
samples	A	5	0.125	0.25	0.025
	B	5	0.25	0.5	0.05
	C	5	0.5	1.0	0.1
	D	7.5	0.125	0.5	0.1
	E	7.5	0.25	1.0	0.025
	F	7.5	0.5	0.25	0.05
	G	10	0.125	1.0	0.05
	H	10	0.25	0.25	0.1
	I	10	0.5	0.5	0.025

#### 2.2.2 Experimental Procedure

In the first phase of the experiment, the true density and porosity of the nine ISCM formulations, as designed in Table 1, were measured. Additionally, each ISCM sphere was placed in a 60 mL transparent glass vial containing 50 mL of RO water. Variations in pH, oxidation–reduction potential (ORP), water absorption, and polyphenol release from green tea leaves were monitored over time. In the second phase, the removal efficiency of lindane from aqueous solution by the nine ISCM formulations was evaluated. An ISCM sphere was added to 20 mL of lindane solution with an initial concentration of 5 mg L^–1^ in a series of 30 mL reaction vials. It should be noted that a higher initial lindane concentration was employed during the development phase to facilitate clearer observation of removal behavior, assessment of degradation kinetics, and identification of transformation products. At a predetermined sampling time period, 6 mL of *n*-hexane was added to each vial for extraction of total lindane mass (including aqueous and leaves sorbed phases). All experiments were performed in duplicate, and average values with associated error ranges were reported.

The Taguchi orthogonal array design was applied to obtain the signal-to-noise (S/N) ratio, which evaluates the effect of the response variables. The S/N ratio, expressed in decibels (dB), was used to indicate lindane removal efficiency, with higher values signifying better performance. The raw data were converted into corresponding S/N ratios using eq 1. Furthermore, the contribution percentage of each parameter was determined through Analysis of Variance (ANOVA).^32–34^

$$\frac{S}{N}(\mathrm{dB})=-10\log\left(\frac{1}{n}\sum_{i=1}^{n}\frac{1}{y_{i}^{2}}\right)$$ 1

The final phase of the experiment examined the release of lindane from lindane spiked contaminated sediments in a static environment, including with and without the application of ISCM (note that the optimal ISCM formulation identified was used in this phase of experiments.). The experiment was conducted in a 6-in. glass tank with a capacity of 3.5 L. The lindane concentration in the contaminated sediment was prepared at 5 mg kg^–1^ (500 g of sediment), with a water volume of 2.5 L. The experimental setup included two groups: a control test (containing only lindane-contaminated sediment) and a remediation test (containing both ISCM and lindane-contaminated sediment). Detailed schematic and actual experimental setups are presented in Figure 1. At each sampling interval, 5 mL of water was collected from 1 cm above the capping layer-water interface from the center line of the short side of the rectangular tank at the position on both sides of the center (the distance between two samples was 10 cm), using a gastight syringe, for both the control and remediation tests. The sample was then extracted for aqueous lindane and its degradation byproduct with *n*-hexane. Polyphenols, pH, and ORP were also measured at the center of the tank.

**Figure 1:**
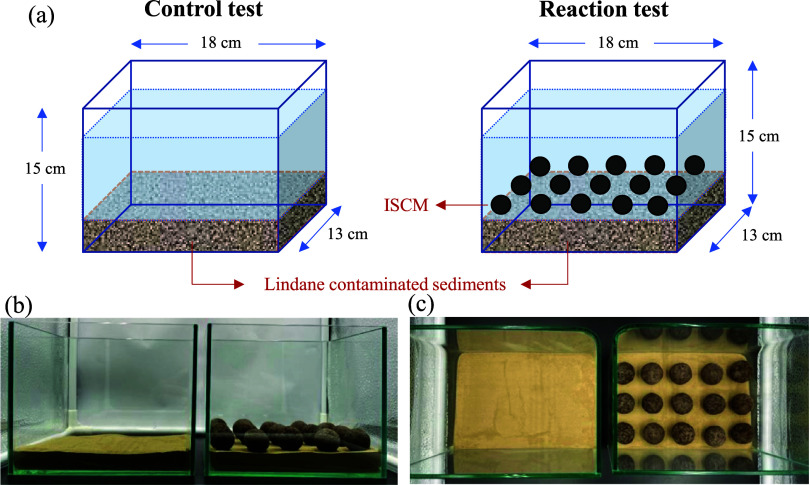
(a) Schematic experimental setup of lindane-contaminated sediments capped by ISCM, (b) side-view, and (c) top-view of reaction tanks.

### 2.3 Analysis

ISCMs were analyzed for porosity and true density using a true density analyzer (Helium pycnometer, Porous Materials Inc., PYC-100A) based on the gas displacement method, with high-purity nitrogen used as the operational gas. Lindane and its degradation byproducts in solution were extracted with *n*-hexane and the extract was qualitatively and quantitatively determined using a gas chromatograph (GC, Agilent 7890A) equipped with a mass spectrometer in electron impact mode (MS, Agilent 5975C)^18^ and separation of compounds was done on a HP-5 ms column. The total phenolic content was determined using the modified Folin-Ciocalteu method established by Kerio et al.^35^ The pH and ORP were measured using a pH/mV/ISE meter (Hanna Instruments HI5222) equipped with a pH combination electrode (Hanna Instruments HI1131B) and an Inlab Redox combination ORP electrode (Mettler Toledo).

## 3 Results and Discussion

### 3.1 Preparation and Characterization of ISCM

The Taguchi method, recognized for determining optimal experimental conditions with minimal experimental runs,^34,36^ was employed with an L9 (3^4^) orthogonal array to evaluate four factors (bentonite, tea leaves, sodium alginate, and pyrite) at three dosage levels each for ISCM preparation (Table 1). The appearance and physical properties of the nine ISCM formulations, along with a bentonite-only control, are presented in Table S1, Supporting Information (SM). It can be seen that as the ratio of pyrite to bentonite increases (i.e., higher pyrite and lower bentonite content, ISCM-C and -E samples), the ISCM shows a darker gray color. However, the amount of tea leaves added did not obviously affect the appearance of the ISCM. The porosity ranged from 7 to 25% (avg. 14.4 ± 5.4%), while all ISCM formulations had real densities between 2.11 and 2.56 g cm^–3^ (avg. 2.23 ± 0.14 g cm^–3^), suggesting a higher density than water and relatively consistent in physical properties across various formulations.

In the subsequent experiment, the ISCMs were placed in 60 mL vials containing 50 mL of RO water to observe changes in appearance, pH, ORP, and polyphenol release over time, as illustrated in Figure 2. All 10 ISCMs (including the control sample with bentonite only) began to absorb water and swell within half a day and reached their maximum swelling by the third day, as compared to the bentonite one, indicating that the presence of tea leaf and pyrite contents did not significantly affect the water absorption and swelling behavior of bentonite. Additionally, the color changes in the vials revealed that ISCMs with higher tea leaf content (ISCM C, F, and I) or a higher tea leaf-to-bentonite ratio (ISCM A, B, and C) released significant amounts of brown tea-polyphenolic substances and then further became darker beyond half a day soaking in water. This is due to an increasing concentration of polyphenols reacting with dissolved iron released from pyrite in the ISCM,^18^ creating the polyphenol/Fe^2+^ reductive system.

**Figure 2:**
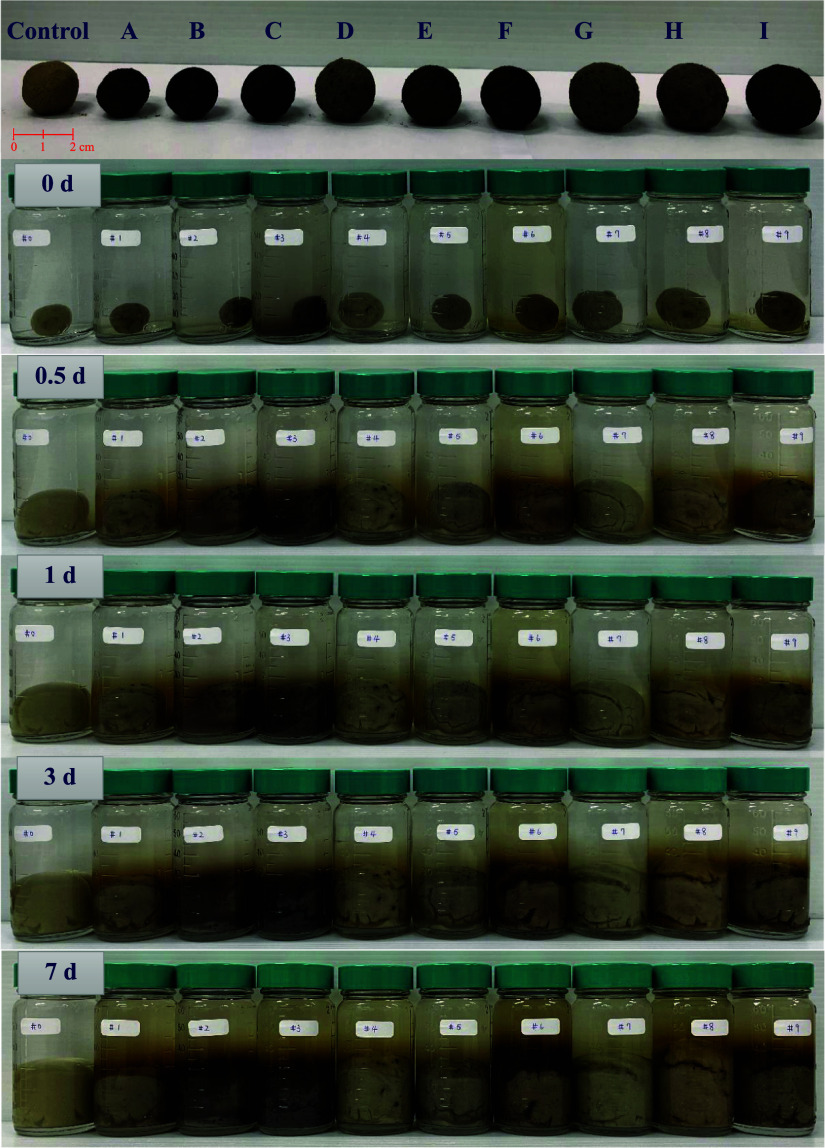
Time-dependent changes in the appearance of various ISCMs immersed in water.

Figure 3 shows the changes over time in (a) pH, (b) ORP, and (c) polyphenol concentration of the nine ISCMs in water. In Figure 3a, all ISCMs initially maintained an alkaline pH environment (between pH 9 and pH 10) in the water. However, observations under the same bentonite mass revealed that ISCMs with higher tea leaf content caused the pH drop to neutral more rapidly. This phenomenon is likely due to the hydrolysis of polyphenols, which releases hydrogen ions under alkaline conditions. When the bentonite mass increased to 10 g, the pH drop became less pronounced. Under alkaline conditions, Fe^2+^ typically precipitates as Fe­(OH)_2_, limiting its reactivity. However, the ISCM developed in this study contains green tea-derived polyphenols that form stable Fe^2+^-phenol complexes, maintaining Fe^2+^ in a chemically active state. These complexes promote Fe^2+^/Fe^3+^ redox cycling, enhancing the system’s reducing power and facilitating lindane dehydrochlorination. Even when Fe­(OH)_2_ forms, it may still contribute to lindane containment through adsorption. These synergistic effects support the ISCM’s role as an effective active capping material for in situ remediation. As shown in Figure 3b, the ORP results indicate that all ISCMs induced a reducing environment in the water within 9 d, with a continued downward trend over time. This effect was particularly evident in ISCM formulations with higher bentonite content, which also maintained an alkaline environment for a longer period. Furthermore, Figure 3c shows that ISCMs with higher tea leaf content exhibited a more pronounced release of total polyphenols over time.

**Figure 3:**
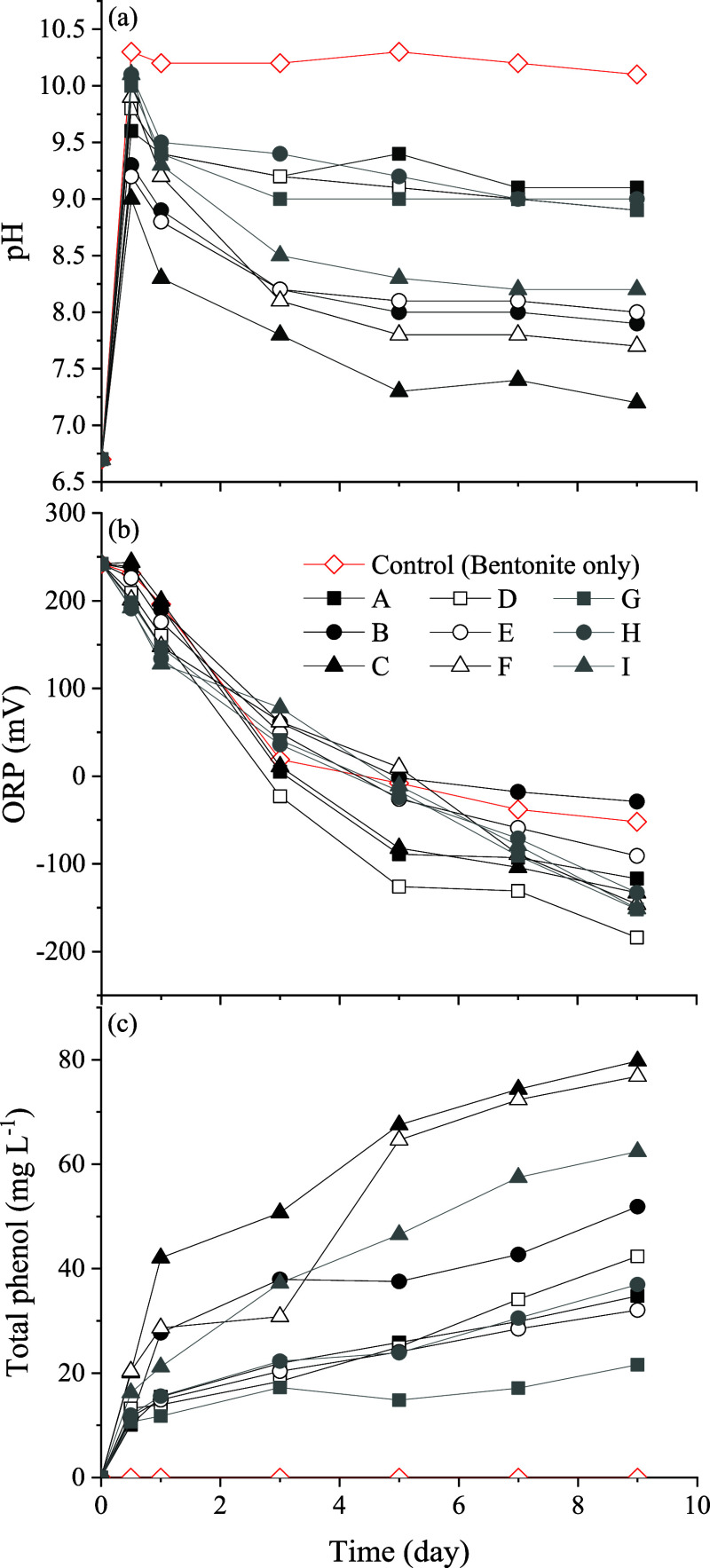
Variation profiles of (a) pH, (b) ORP, and (c) polyphenol concentration in water containing different ISCMs.

### 3.2 Degradation Experiment of Aqueous-Phase Lindane by ISCM

Based on the experimental results from Section 3.1, all nine ISCM formulations exhibited good water absorption and swelling properties, capability of maintaining an alkaline environment, and continuous release of polyphenolic compounds to initiate polyphenol/Fe^2+^ reactions, ultimately creating a reducing environment. Consequently, further evaluation was conducted to examine the capacity of the nine ISCMs in degrading lindane in aqueous solution. Wang et al.^21^ reported that tea leaves can adsorb OCPs; therefore, to avoid underestimating the ISCM’s reductive degradation performance, lindane was extracted from both the solid phase (including any adsorbed on tea leaves) and the liquid phase. Figure 4a illustrates the removal of lindane from water over time in the presence of different ISCMs. The results indicate that all ten ISCM formulations (including the control sample with bentonite only) effectively removed lindane within 9 days. However, the lindane removal by the control group was primarily due to alkaline hydrolysis with formation of byproduct PCCHe, along with trace amounts of TCBs,^5,18^ as evidenced in Figure 4b by mass spectrometry analysis of the solution from the control group on day 9. Note that based on GC-MS analysis, the predominant TCBs identified by retention time and mass spectra were 1,2,4-trichlorobenzene and 1,3,5-trichlorobenzene. However, no alkaline hydrolysis byproducts were detected in the other nine ISCM formulations (see Figure 4c, with ISCM-C as an example).

**Figure 4:**
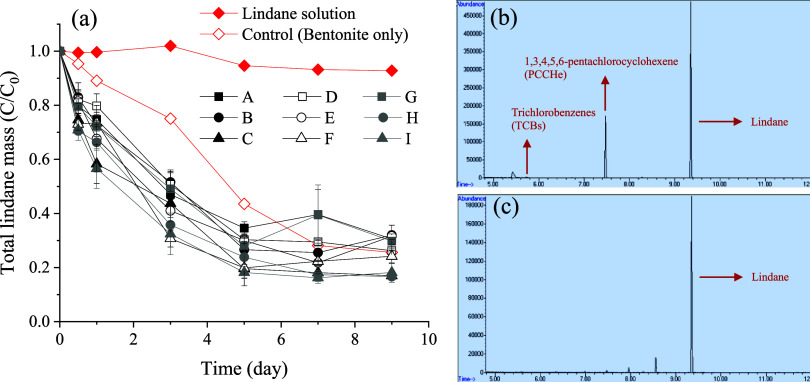
(a) Degradation of total lindane mass in the reaction system by different ISCMs over time in aqueous phase experiments, along with the mass spectrometry qualitative analysis from (b) the control test and (c) ISCM-C in water after 9 days of reaction.

The relevant impact of the experimental parameters on lindane removal was evaluated using ANOVA. The objective was to achieve the highest possible lindane removal efficiency. Therefore, the Signal-to-Noise (S/N) ratio characteristic used in this study was set to “The Larger the Better.” ^34,36^ The S/N ratio analysis was applied to distinguish the influence of various factors, including the amounts of bentonite, tea leaves, sodium alginate, and pyrite and the corresponding S/N ratio values under nine experimental conditions are presented in Table S1 (SM). The S/N ratio increased across the nine experiments with increasing lindane removal efficiency. The highest lindane removal efficiency (83.3%) and the highest S/N ratio (38.42 dB) were observed in ISCM-H. Similarly, the lowest lindane removal efficiency (68.0%) and the lowest S/N ratio (36.65 dB) were found in ISCM-B.

Table 2 presents the average response values for the S/N ratio and lindane removal efficiency at each level. The S/N ratio for the bentonite addition factor was highest at level 3 (10 g), reaching 37.87 dB, while levels 1 and 2 showed similarly lower S/N ratios. The average removal efficiency decreased from 78.46% at level 3 to 72.64 and 73.49% at levels 2 and 1, respectively. A similar trend was observed for the sodium alginate addition factor, with the S/N ratio reaching its highest value (38.05 dB) at level 3 (0.1 g), while levels 1 and 2 exhibited similarly lower S/N ratios. The average removal efficiency induced by sodium alginate factor decreased from 80.03% at level 3 to 71.36 and 73.21% at levels 2 and 1, respectively (see Table 2). These results indicate that higher additions of bentonite and sodium alginate are more favorable for lindane removal by tea leaves ISCM. Furthermore, as the amount of tea leaves increased, both the S/N ratio and the average lindane removal efficiency also increased, likely because higher amounts of tea leaves provide sufficient polyphenols to drive the reductive reaction. However, when examining the effect of pyrite addition, it was found that the S/N ratio was highest (37.61 dB) at the lowest pyrite addition level (0.25 g) and lowest (37.34 dB) at the highest addition level (10 g). In the reaction system, as the amount of pyrite increased, lindane removal efficiency decreased (from 76.19% at level 1 to 73.87% at level 3). Although this observed impact in this study was not significant, Wang et al.^18^ suggested that this could be due to excessive Fe^2+^ forming green rust complexes, which may disrupt the reductive environment necessary for the dechlorination of lindane in the polyphenol/Fe^2+^ system.

**Table 2:** Averaged Responses of S/N Ratio of Lindane Removal and Averaged Lindane Removal Efficiency by ISCM at Each Level

	factors			
level	bentonite	tea leaves	pyrite	sodium alginate
**S/N ratio (dB)** ^a^				
1	37.29	37.04	37.61	37.26
2	37.22	37.26	37.42	37.06
3	37.87	38.08	37.34	38.05
delta^b^	0.65	1.04	0.28	0.99

**Averaged Lindane removal efficiency (%)**				
1	73.49	71.15	76.19	73.21
2	72.64	73.24	74.52	71.36
3	78.46	80.21	73.87	80.03
delta^b^	5.82	9.06	2.32	8.67

a S/N ratio at each level = (S/N_1_ + S/N_2_ + S/N_3_)/3, where S/N_1_, S/N_2_, and S/N_3_ are S/N ratios of individual factor at level 1, 2, and 3, respectively.

b Delta represents the difference between the maximum and minimum S/N ratio or averaged Lindane removal efficiency for each factor.

To further determine the contribution of each factor in the ISCM system, ANOVA was conducted, and the results are presented in Table S2 (SM). The contribution rate was calculated using eq 2:^34^

$$\mathrm{Contribution}\;\mathrm{rate}\;(\%)=\frac{{\mathrm{SS}}_{A}}{{\mathrm{SS}}_{T}}\times 100\%$$ 2

where SS_A_ represents the sum of squares for a particular variable (e.g., factor A), and SS_T_ represents the total sum of squares.

In Table S2 (SM), the percentage contributions of each factor to lindane removal are as follows: tea leaves (41.5%), sodium alginate (38.0%), bentonite (17.7%), and pyrite (2.8%). These results indicate that the addition of tea leaves is the most significant factor for lindane removal by tea leaves ISCM, whereas pyrite has the least impact. Based on the predicted optimal conditions, bentonite at level 3 (10 g), tea leaves at level 3 (0.5 g), pyrite at level 1 (0.25 g), and sodium alginate at level 3 (0.1 g) were evaluated. The treatment efficiency predicted by the Taguchi method was 90.3% (see Table S3 (SM) for detailed experimental conditions and calculations). A confirmation experiment was conducted to validate the predicted result and assess the significance of input factors in determining the optimal operational conditions. The experimentally obtained efficiency was close to the predicted value, with a lindane degradation rate of 89.6% under optimized experimental conditions. These results suggest that the prepared tea leaves ISCM reveals the potential application for lindane removal.

### 3.3 Simulated Field Scale Experiments on the Remediation of Lindane-Contaminated Sediments Using ISCM

ISCMs were prepared using the optimal material composition determined by the Taguchi method, and their effectiveness in remediating lindane-contaminated sediments was evaluated through simulated applications. Figure 5 shows the setup of the sediment-phase experiment and the arrangement of ISCM placed above the sediment. ISCM was applied with a single layer, and consistent spacing between each unit (ISCM diameter approximately 2 cm, with 1.5 cm between units, totaling 15 units). This arrangement aimed to observe the transformation of ISCM from a dry to a swollen state upon water absorption. Excessively dense placement could lead to overcompression during swelling, introducing uncontrollable variables. Figure 5 also presents photographs illustrating the temporal changes in the reaction tank’s appearance during the application of ISCM for the remediation of lindane-contaminated sediment. In the control group with only lindane-contaminated sediment, the system showed unchanged throughout the 15-day reaction period, except for minor disturbance and turbidity during the initial water addition. In contrast, in the ISCM remediation group, the ISCMs absorbed water and swelled over time, filling the gaps between ISCM units by day one and effectively stabilizing and covering the sediment. As the reaction progressed, the ISCM group exhibited a gradual appearance of tea-brown polyphenols from the capping layer, with the color turning dark green by day 9. This color change is consistent with previous experimental results (Figure 2) and is likely due to the tea polyphenol/Fe^2+^ (bluish-green) or Fe^3+^ (ochre) complex formation at alkaline pH.^18^

**Figure 5:**
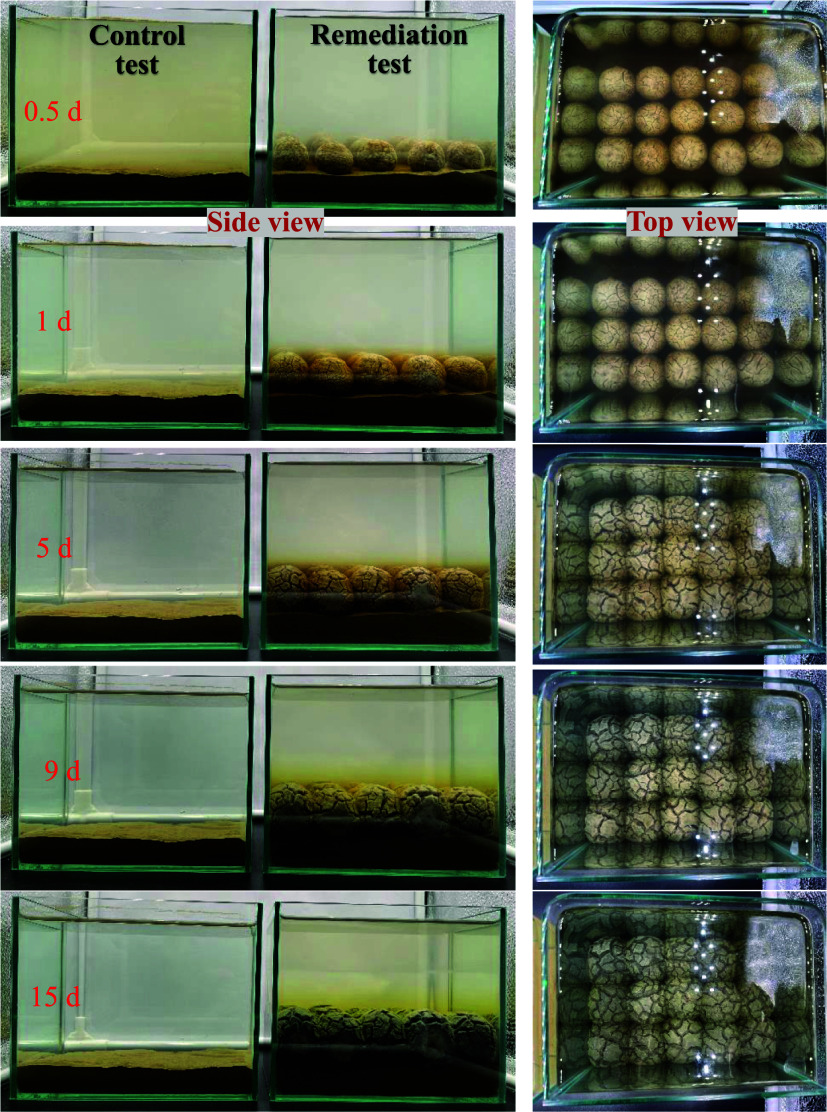
Setup of a simulated field-scale experiment for remediating lindane-contaminated sediments using ISCM, along with the observed changes in ISCM appearance over time during the reaction.

The variation of lindane and polyphenol concentrations in the water and the changes in pH and ORP during the course of the reaction with lindane-contaminated sediments covered by ISCM are illustrated in Figure 6a,b, respectively. In the control group, the contaminated sediment exhibited a neutral pH of approximately 6.5 over the 15-day period, while the ORP remained around 220 mV. The contaminated sediment gradually released lindane into the water, reaching a steady concentration of approximately 0.048 ± 0.001 mg L^–1^ on day 9. In contrast, in the ISCM remediation group, no lindane release was detected over the 15 days, indicating that ISCM effectively stabilized the lindane beneath the capping layer. In previous studies, Gu et al.^37^ used bentonite, Illite, and zeolite as capping materials to stabilize sediment nutrients and reduce nitrogen compound release, whereas Zhang et al.^38^ employed modified biochar to stabilize and adsorb Cu^2+^ and 4-chlorophenol from sediment. Both techniques demonstrated that in situ capping can chemically and physically inhibit the release of contaminants from contaminated sediments. In addition, several sediment capping systems have been developed to target chlorinated organic pollutants, particularly through redox-active or sorptive materials. For example, McDonough et al.^39^ demonstrated the use of activated carbon for the sequestration of PCBs in field-scale sediment caps, emphasizing sorption as the primary mechanism. More recently, studies have explored Fe-amended reactive caps for promoting reductive dechlorination of halogenated compounds,^40,41^ aligning closely with the mechanism employed in the present ISCM. The polyphenol-Fe^2+^ system described here builds on these strategies by facilitating electron transfer through Fe^2+^/Fe^3+^ redox cycling, thus enhancing the degradation of lindane in addition to its containment. This dual functionality offers a promising advancement in active cap design for in situ remediation of persistent organic pollutants. Moreover, a study by Wang and Liang^18^ investigating lindane degradation kinetics and mechanisms in aqueous systems using a green tea leaf/iron system identified two primary pathways: alkaline hydrolysis and reductive dechlorination. Table S4 (SM) summarizes the degradation products formed via these pathways, along with their respective LC_50_ (median lethal concentration) values. The data show that all identified degradation products have significantly higher LC_50_ values (ranging from 2.1 to 57.0 mg L^–1^) compared to the parent compound lindane (0.087 mg L^–1^), indicating markedly lower toxicity. These values reflect a toxicity reduction of approximately 24- to 650-fold relative to lindane. Building on this, the ISCM developed in the present study employs a reductive reaction mechanism that effectively suppresses the release of lindane. Over a 15-day period, no lindane was detected in the ISCM-treated system, compared to 0.05 mg L^–1^ in the control without ISCM. By enabling controlled release, the tea polyphenol/Fe^2+^ complex within the ISCM actively participated in lindane degradation. The optimized formulation, comprising 10 g bentonite, 0.5 g tea leaves, 0.5 g pyrite, and 0.1 g sodium alginate, achieved 90% lindane degradation in prior aqueous-phase experiments. These results suggest that the ISCM capping layer not only facilitated in situ lindane degradation but also effectively prevented its migration into surrounding water.

**Figure 6:**
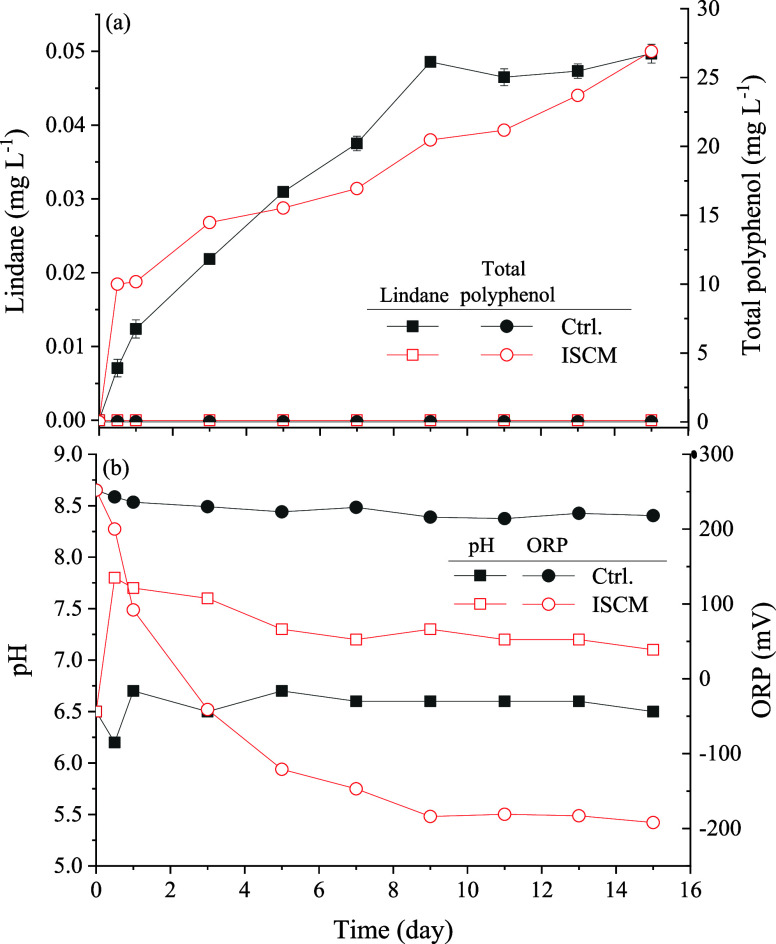
(a) Release of lindane and polyphenol concentrations in the water with reaction time and (b) the change of pH and ORP in the reaction system with lindane-contaminated sediments covered by ISCM (Lindane conc. = 5 mg kg^–1^).

Additionally, during the reaction, polyphenols were released into the water, with a concentration above the ISCM capping layer reaching approximately 27 mg L^–1^ on day 15. This indicates that ISCM can continuously release polyphenols, maintaining sufficient polyphenol concentration near the ISCM capping layer in the static water environment. Pertaining to pH and ORP changes, the water initially exhibited a slightly alkaline level (pH = 7.5 to 8.0 from days 0 to 3), then gradually decreased to neutral (around 7.3) by day 15. The ORP values also decreased over time, indicating a reducing environment with approximately −200 mV. These water qualities may suggest that the internal environment of the ISCM capping layer is likely more alkaline and reducing, with a limited effect on the pH, outside the capping layer. This remediation condition is favorable for the polyphenol/Fe^2+^ reaction occurred within the capping layer. Based on the results of preliminary remediation experiments, the ISCM developed in this study demonstrated excellent water absorption and swelling properties. Additionally, the polyphenol/Fe^2+^ reductive reaction within the sediment capping layer effectively stabilized lindane-contaminated sediment, preventing pollutant diffusion and potentially enhancing pollutant degradation. An acute toxicity test using juvenile carp (*Cyprinus carpio*) determined the LC_50_ of green tea polyphenols to be 217.5 mg L^–1^ (Table S5, SM). Given that the measured concentration above the ISCM layer after 15 d was approximately 27 mg L^–1^, the release of polyphenols is unlikely to pose significant ecological risks under the conditions tested.

## 4 Conclusions

In this study, a novel in situ capping material was developed using polyphenol-rich tea leaves, bentonite, sodium alginate, and pyrite to remediate lindane-contaminated sediments. Experimental results showed that ISCM effectively absorbs water, swells, and forms a stable reactive capping layer, providing both physical isolation and active reductive degradation of lindane. Employing the Taguchi method facilitated optimization of the ISCM composition, resulting in a formulation that maximizes remediation efficiency. Based on the signal-to-noise ratio and lindane removal efficiency analyzed by ANOVA, tea leaves contributed most, followed by sodium alginate, bentonite, and pyrite. Beyond stabilizing sediments, the ISCM promotes pollutant breakdown through the reducing properties of tea polyphenols and Fe^2+^ released by pyrite. Aqueous- and sediment-phase experiments demonstrated significant lindane degradation, with optimized formulations outperforming conventional capping materials. These findings highlight ISCM as a promising, sustainable, and green technology for in situ sediment remediation, underscoring the key role of natural polyphenols as multifunctional agents in environmental engineering.

## Supplementary Material



## Notes

Physical properties and corresponding S/N ratios in the lindane degradation experiment; ANOVA results for degradation of lindane by ISCM; comparison of prediction and confirmation experiment for degradation of Lindane by ISCM; toxicity LC50 values for lindane and its degradation products; and acute toxicity analysis results of various substances (PDF)
