# Supplementary material for: Evaluation of Green Tea Leaves as an In Situ Capping Material for the Remediation of Lindane-Contaminated Sediments
Source: ACS Omega. 2025 Jul 29;10(31):34321–30. doi: 10.1021/acsomega.5c01779 (PMC12355268; doi:10.1021/acsomega.5c01779)
Supplement: Supplementary file 1 [file ao5c01779_si_001.pdf]

Supplementary Materials

Evaluation of Green Tea Leaves as an In-Situ Capping Material for the Remediation of Lindane Contaminated Sediments

Chi-Wei Wang[a,b], Chenju Liang[a,*]

[a] Department of Environmental Engineering, National Chung Hsing University

250 Kuo-kuang Road, Taichung 402, Taiwan

[b] Department of Environmental Engineering, Da-Yeh University

168 University Road, Dacun, Changhua 515006, Taiwan

*Corresponding author. Tel.: +886-4-22856610; Fax: +886-4-22856610

Chenju Liang email address: cliang@nchu.edu.tw

Chi-Wei Wang email address: chwang@mail.dyu.edu.tw

Contents

Table S1. Physical properties and corresponding S/N ratios of different ISCM formulations employed in the lindane degradation experiment.

Table S2. ANOVA results for degradation of lindane by ISCM in the aqueous phase experiment.

Table S3. Comparison of prediction and confirmation experiment for degradation of Lindane by ISCM.

Table S4. The toxicity LC_{50} values for lindane and its degradation products.

Table S5. Acute toxicity analysis results of various substances.

Table S1. Physical properties and corresponding S/N ratios of different ISCM formulations employed in the lindane degradation experiment.

Sample	Photo	Mass (g)	Volume (cm^3)	Porosity (%)	Real density (g cm^{-3})	S/N Ratio
Control (bentonite only)	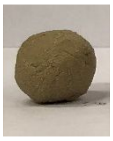	2.57	1.11	16.5	2.312 ± 0.032	Not available
A	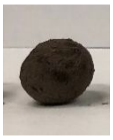	2.60	1.20	7.2	2.168 ± 0.021	36.83
B	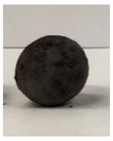	2.89	1.34	12.9	2.125 ± 0.001	36.65
C	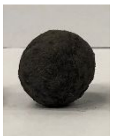	3.29	1.38	20.1	2.383 ± 0.024	38.38
D	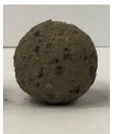	4.27	1.86	15.1	2.277 ± 0.016	37.35
E	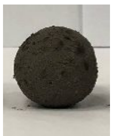	4.32	1.71	25.2	2.526 ± 0.017	36.70
F	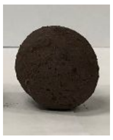	4.28	1.95	10.8	2.191 ± 0.005	37.59
G	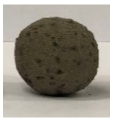	5.71	2.63	12.8	2.172 ± 0	36.93
H	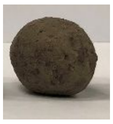	5.51	2.61	10.6	2.111 ± 0.007	38.42
I	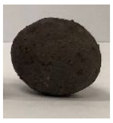	5.96	2.81	15.3	2.120 ± 0	38.26

Table S2. ANOVA results for degradation of lindane by ISCM in the aqueous phase experiment.

Factors	DOF [a]	SS	Contribution (%)
Bentonite	2	0.77	17.7
Tea leaves	2	1.80	41.5
Pyrite	2	0.12	2.8
Sodium alginate	2	1.65	38.0
Total	8	4.33	100.0

[a] DOF: degree of freedom.

Table S3. Comparison of prediction and confirmation experiment for degradation of Lindane by ISCM.

	Lindane degradation efficiency	
Optimal levels of process parameters	Predicted optimal values (%)[a]	Average of confirmation experiment (%)[b]
Bentonite 10 g (A)<br>Tea leaves 0.5 g (B)<br>Pyrite 0.25 g (C)<br>Sodium alginate 0.1 g (D)	90.3	89.6

Note: [a] Predicted optimal values = $\bar{y} + (A - \bar{y}) + (B - \bar{y}) + (C - \bar{y}) + (D - \bar{y})$, where: $\bar{y}$ is the averaged lindane removal efficiency of the total experimental results; A, B, C, and D are averaged lindane removal efficiencies of individual factor under optimal conditions. [b] Averaged lindane removal efficiency obtained from the confirmation experiment.

Table S4. The toxicity LC_{50} values for lindane and its degradation products.

Compound	Chemical formula	Pathway	Species	LC_{50}
Lindane	$C_6H_6Cl_6$		Fathead minnow	0.087 mg/L for 96 h
1,3,4,5,6-Pentachlorocyclohexene	$C_6H_5Cl_5$	Alkaline hydrolysis	n.a.	n.a.
2,3,4,5,-Tetrachlorocyclohexene	$C_6H_6Cl_4$	Reductive dechlorination	n.a.	n.a.
1,2,4-Trichlorobenzene	$C_6H_3Cl_3$	Alkaline hydrolysis	Fathead minnow	2.8 mg/L for 96 h
1,2,3-Trichlorobenzene			Flagfish	2.1 mg/L for 96 h
1,3,5-Trichlorobenzene			Guppy	3.3 mg/L for 14 d
1,2-Dichlorobenzenes	$C_6H_4Cl_2$	Reductive dechlorination	Fathead minnow	57.0 mg/L for 96 h
1,3-Dichlorobenzenes			Fathead minnow	12.7 mg/L for 96 h
1,4-Dichlorobenzenes			Fathead minnow	33.7 mg/L for 96 h
Chlorobenzene	C_6H_5Cl	Reductive dechlorination	Fathead minnow	16.9 mg/L for 96 h
Benzene	C_6H_6	Reductive dechlorination	Fathead minnow	24.6 mg/L for 96 h

Note: LC_{50} value of each compound quoted from https://pubchem.ncbi.nlm.nih.gov/; n.a.: not available.

Table S5. Acute toxicity analysis results of various substances.

Species	Initial Conc. (mg/L)	LC_{50} (mg/L)	Analysis mode[(a)]
Green tea	750[(b)]	217.50	Spearman-Karber
$FeSO_4 \cdot 7H_2O$	250	177.68	Spearman-Karber
Bentonite	6,000	n.a.	n.a.
H^+	0.2	0.18	Graphical
OH^-	42.5	24	Spearman-Karber

Note: [(a)] In accordance with the procedure established by USEPA; [(b)] The concentration was defined by total polyphenol.; n.a.: not available.

Analytical procedure:

"The acute toxicity test was conducted following the guidelines of the Taiwan National Institute of Environmental Analysis (NIEA)[1]. The test organism, juvenile carp (*Cyprinus carpio*), was exposed to five serial dilutions (20%, 40%, 60%, 80%, and 100%) of the test solution for 96 h without feeding, and mortality was recorded at regular intervals. A control group using RO water was also included. LC_{50} values were estimated using standard statistical methods, including the Graphical, Spearman-Karber, and Trimmed Spearman-Karber methods, as appropriate, based on data distribution and mortality patterns, in accordance with NIEA protocols adapted from the United States Environmental Protection Agency (USEPA)[2]."

References:

(1) NIEA, 2005. Aquatic Acute Toxicity Test Method – A Static Cyprinus Carpio Toxicity Test. Taiwan National Institute of Environmental Analysis (NIEA). Method B904.11B.

(2) USEPA, 2002. Methods for Measuring the Acute Toxicity of Effluents and Receiving Waters to Freshwater and Marine Organisms. United States Environmental Protection Agency (USEPA), Washington DC. EPA/821/R-02/012.
